# (3,5-Di-*tert*-butyl-2-eth­oxy­benzyl­idene)[2-(3,5-di-*tert*-butyl-1*H*-pyrazol-1-yl)eth­yl]amine

**DOI:** 10.1107/S160053681203231X

**Published:** 2012-07-21

**Authors:** Lara C. Spencer, Ilia A. Guzei, Michael K. Ainooson, James Darkwa

**Affiliations:** a Department of Chemistry, University of Wisconsin-Madison, 1101 University Ave, Madison, WI 53706, USA; b Department of Chemistry, University of Johannesburg, Auckland Park Kingsway Campus, Johannesburg 2006, South Africa

## Abstract

The angles within the benzene ring in the title compound, C_30_H_49_N_3_O, ranging from 116.34 (16) to 124.18 (16)°, reflect the presence of electron-donating and electron-withdrawing substituents. The angles at the two electron-donating *tert*-butyl substituents are smaller than 120°, at the electron-withdrawing eth­oxy substituent larger than 120°, and at the imine substituent equal to 119.59 (16)°. The latter does not reflect the electron-donating nature of the imine group due to the presence of other substituents.

## Related literature

For information on (pyrazol-1-yl)imine ligands that feature phenol in cobalt and palladium complexes see: Ainooson (2010 ▶); Boltina *et al.* (2012 ▶). Geometrical parameters were checked with *Mogul* (Bruno *et al.*, 2002 ▶). Related compounds were found in the Cambridge Structural Database (Allen, 2002 ▶).
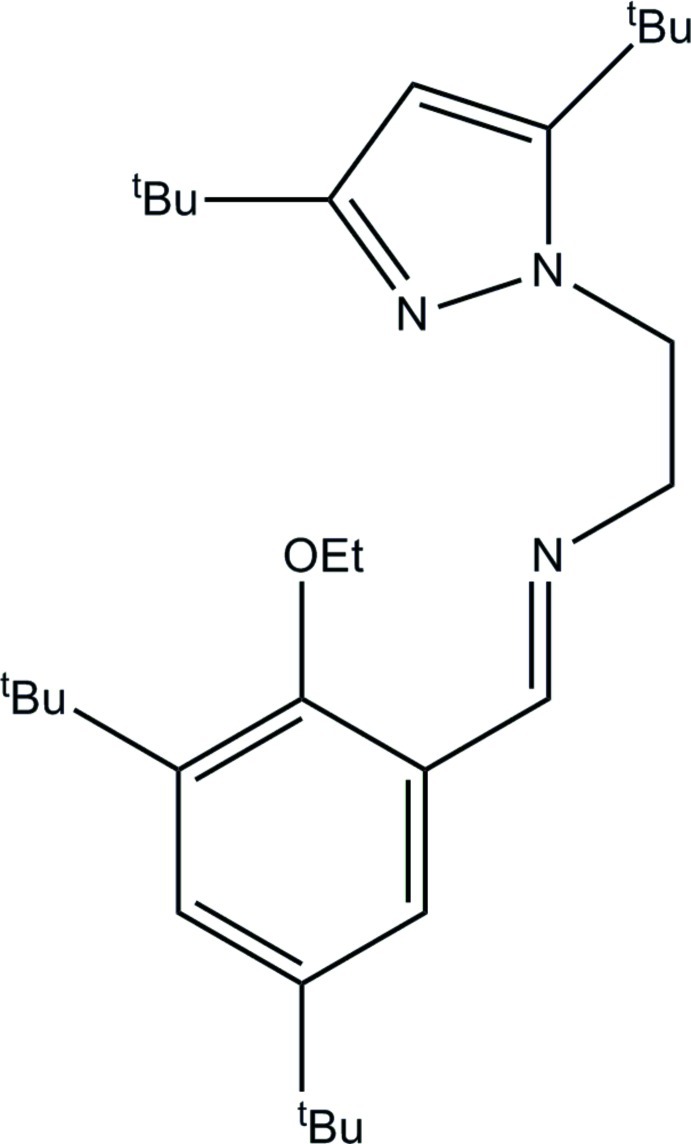


## Experimental

### 

#### Crystal data

C_30_H_49_N_3_O
*M* *_r_* = 467.72
Triclinic, 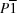

*a* = 10.9220 (3) Å
*b* = 11.6071 (4) Å
*c* = 12.0283 (4) Å
α = 78.452 (2)°
β = 82.775 (2)°
γ = 79.146 (2)°
*V* = 1461.11 (8) Å^3^
*Z* = 2
Cu *K*α radiation
μ = 0.49 mm^−1^
*T* = 100 K
0.12 × 0.10 × 0.09 mm

#### Data collection

Bruker APEXII CCD diffractometer
Absorption correction: multi-scan (*SADABS*; Bruker, 2003) ▶ *T*_min_ = 0.944, *T*_max_ = 0.958
25868 measured reflections
5197 independent reflections
3560 reflections with *I* > 2σ(*I*)
*R*_int_ = 0.058

#### Refinement

*R*[*F* ^2^ > 2σ(*F* ^2^)] = 0.046
*wR*(*F* ^2^) = 0.123
*S* = 1.00
5197 reflections
320 parameters
H-atom parameters constrained
Δρ_max_ = 0.18 e Å^−3^
Δρ_min_ = −0.19 e Å^−3^

### 

Data collection: *APEX2* (Bruker, 2007) ▶; cell refinement: *SAINT* (Bruker, 2007) ▶; data reduction: *SAINT*; program(s) used to solve structure: *SHELXTL* (Sheldrick, 2008 ▶); program(s) used to refine structure: *SHELXTL* and *FCF_filter* (Guzei, 2007 ▶); molecular graphics: *DIAMOND* (Brandenburg, 1999 ▶); software used to prepare material for publication: *SHELXTL*, *publCIF* (Westrip, 2010 ▶) and *modiCIFer* (Guzei, 2007 ▶).

## Supplementary Material

Crystal structure: contains datablock(s) global, I. DOI: 10.1107/S160053681203231X/bh2447sup1.cif

Structure factors: contains datablock(s) I. DOI: 10.1107/S160053681203231X/bh2447Isup2.hkl

Supplementary material file. DOI: 10.1107/S160053681203231X/bh2447Isup3.cml

Additional supplementary materials:  crystallographic information; 3D view; checkCIF report

## Figures and Tables

**Fig. 1.**
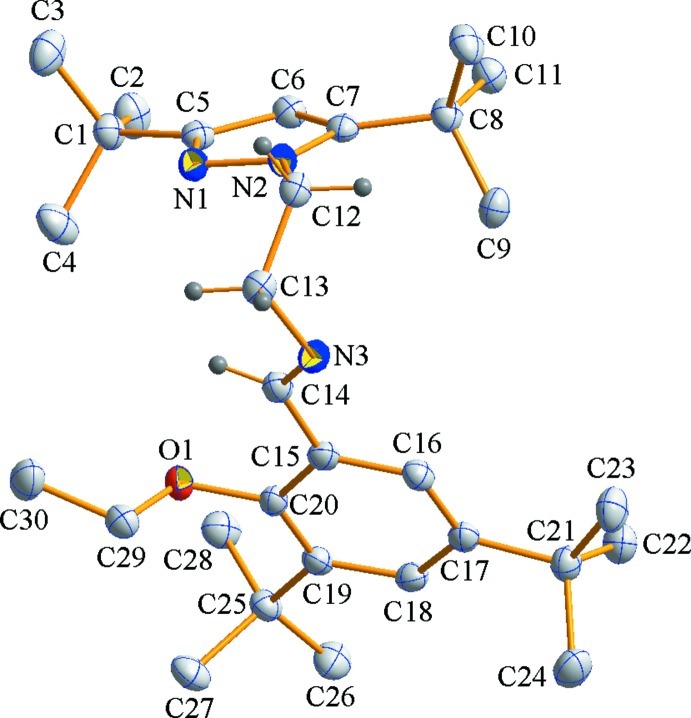
Molecular structure of I (Brandenburg, 1999). The thermal ellipsoids are shown at 50% probability level. The hydrogen atoms on C12, C13 and C14 are shown to clarify the location of the imine double bond. All other hydrogen atoms are not shown.

## supplementary crystallographic information

### Comment

(Pyrazol-1-yl)imine ligands that feature phenol have recently been used as ligands in preparing cobalt (Ainooson, 2010) and palladium (Boltina *et al*., 2012) complexes, but the phenol proton reacts with cobalt and palladium to form an undesirable HCl by-product. To avoid the formation of HCl in such reactions, we have studied compounds where the phenol is replaced with an alkyl or aryl group. One example of such a potential ligand precursor is the ethoxy derivative, I, the title compound, reported herein.

The *Mogul* check of I confirmed that the geometrical parameters are typical except for the C12—N2—N1 angle and the C12—N2—C7 angle (Bruno *et al.*, 2002). A search of the Cambridge Structural Database (CSD; Allen, 2002) yielded 45 related compounds that have the pyrazole—C—C—N—C—benzene backbone. In these related compounds the angle comparable to the C12—N2—N1 angle in I had an average of 118 (6)° and a range of 101.62 to 123.99°. The 115.52 (14)° value for the C12—N2—N1 angle in I is within the range and within the standard deviation of the average for the related compounds, and thus should not be considered atypical. For the 45 related compounds the comparable C7—N2—C12 angle had an average of 126 (5)° with a range of values from 103.56 to 133.10°. The value for I of 132.37 (15)° is within the range of values for the related compounds.

The angles of the C15···C20 benzene ring range from 116.34 (16) to 124.18 (16)° deviating from the ideal 120° angle due to the presence of electron donating and electron withdrawing substituents. The two *tert*-butyl groups on atoms C17 and C19 are electron donating and the angles at these two carbon atoms in the benzene ring are expectedly smaller than the ideal 120° at 117.23 (17) and 116.34 (16)°, respectively. The ethoxy group at C20 is electron withdrawing and thus the angle at its *ipso* carbon atom is expected to exceed 120°, and indeed the angle measures 121.38 (17)°. The imine group at C15 is expected to be an electron donor with its *ipso* angle spanning less than 120°. This expectation is supported by a CSD search of monosubstituted benzene rings bearing an imine group: in 234 crystals the angle of interest averaged over 304 entries is 118.9 (9)°. In the case of I, however, the ring angle at C15 is very close to 120 at 119.59 (16)°. This is likely due to the presence of other substituents.

### Experimental

A mixture of 3,5-di-tertiarybutyl-2-ethoxybenzaldehyde (0.40 g, 1.50 mmol), 2-(3,5-di-tertiarybutylpyrazol-1-yl)ethylamine hydrochloride (0.39 g, 1.70 mmol) and excess anhydrous magnesium sulfate (0.40 g, 3.30 mmol) in ethanol (20 ml) was refluxed for 4 h. The yellow filtrate obtained after filtration was evaporated to a yellow oil, which was re-dissolved in dichloromethane (20 ml) and layered hexane (10 ml) and kept at 269 K for 3 days, to afford light yellow crystals. Yield: 0.67 g (95%). ^1^H (CDCl_3_) δ: 1.23 (s, 9H, ^t^Bu); 1.28 (s, 9H, ^t^Bu); 1.35 (s, 9H, ^t^Bu); 1.36 (s, 9H, ^t^Bu); 1.47 (t, 3H,^3^*J*_HH_= 6.9 Hz, *CH_3_*CH_2_O); 3.74 (q, 2H, ^3^*J*_HH_= 6.9 Hz ^2^*J*_HH_ = 6.9 Hz, O*CH_2_*CH_3_); 4.14 (t, 2H, ^3^*J*_HH_ = 5.7 Hz, CH_2_); 4.47 (t, 2H, ^2^*J*_HH_ = 6.3 Hz, CH_2_); 5.71 (s, 1H, pz-H); 7.37 (d, 1H, ^4^*J*_HH_= 2.7 Hz, Ar—H); 7.69 (d, 1H, ^4^*J*_HH_ = 2.4 Hz, Ar—H); 8.42 (s, 1H, CH=N); ^13^C{^1^H} (CDCl_3_) δ: 15.3; 30.5; 30.6; 30.9; 31.2; 31.4; 31.9; 34.6; 35.1; 50.7; 61.7; 72.3; 98.9; 122.5; 126.8; 128.9; 141.9; 145.6; 151.6; 156.8; 159.9; 160.4. IR (Diamond ATR, cm^-1^): 1631*υ*(CH=N), 1320 *υ*(C—O). HRMS (ESI) (m/*z*) [*M*^+^+H^+^]: Anal. Calcd. for C_30_H_50_N_3_O: 468.3954. Found: 468.3976.

### Refinement

All H-atoms were placed in idealized locations and refined as riding with appropriate thermal displacement coefficients: *U*_iso_(H) = 1.2 times *U*_eq_(bearing atom) for C(*sp*^2^)-H and C(*sp*^3^)-2H hydrogen atoms and *U*_iso_(H) = 1.5 times *U*_eq_(bearing atom) for C(*sp*^3^)-3H hydrogen atoms. Default effective *X*—H distances for *T* = 100 K were used: C(*sp*^2^)-H = 0.95, C(*sp*^3^)-2H = 0.99, C(*sp*^3^)-3H = 0.98 Å.

### Crystal data

**Table:**

C_30_H_49_N_3_O	*Z* = 2
*M**_r_* = 467.72	*F*(000) = 516
Triclinic, *P*1	*D*_x_ = 1.063 Mg m^−^^3^
Hall symbol: -P 1	Cu *K*α radiation, λ = 1.54178 Å
*a* = 10.9220 (3) Å	Cell parameters from 3414 reflections
*b* = 11.6071 (4) Å	θ = 3.8–67.9°
*c* = 12.0283 (4) Å	µ = 0.49 mm^−^^1^
α = 78.452 (2)°	*T* = 100 K
β = 82.775 (2)°	Block, colourless
γ = 79.146 (2)°	0.12 × 0.10 × 0.09 mm
*V* = 1461.11 (8) Å^3^	

### Data collection

**Table:**

Bruker APEXII CCD diffractometer	5197 independent reflections
Radiation source: fine-focus sealed tube	3560 reflections with *I* > 2σ(*I*)
Graphite monochromator	*R*_int_ = 0.058
0.50° ω and 0.5 ° φ scans	θ_max_ = 69.4°, θ_min_ = 3.8°
Absorption correction: multi-scan (*SADABS*; Bruker, 2003)	*h* = −13→13
*T*_min_ = 0.944, *T*_max_ = 0.958	*k* = −13→13
25868 measured reflections	*l* = −14→14

### Refinement

**Table:**

Refinement on *F*^2^	Primary atom site location: structure-invariant direct methods
Least-squares matrix: full	Secondary atom site location: difference Fourier map
*R*[*F*^2^ > 2σ(*F*^2^)] = 0.046	Hydrogen site location: inferred from neighbouring sites
*wR*(*F*^2^) = 0.123	H-atom parameters constrained
*S* = 1.00	*w* = 1/[σ^2^(*F*_o_^2^) + (0.068*P*)^2^ + 0.050*P*] where *P* = (*F*_o_^2^ + 2*F*_c_^2^)/3
5197 reflections	(Δ/σ)_max_ < 0.001
320 parameters	Δρ_max_ = 0.18 e Å^−^^3^
0 restraints	Δρ_min_ = −0.19 e Å^−^^3^
0 constraints	

### Fractional atomic coordinates and isotropic or equivalent isotropic displacement parameters (Å^2^)

**Table:**

	*x*	*y*	*z*	*U*_iso_*/*U*_eq_	
O1	0.34819 (11)	0.66011 (10)	0.25603 (10)	0.0235 (3)	
N1	0.28108 (14)	0.73254 (13)	−0.14877 (12)	0.0232 (4)	
N2	0.15493 (14)	0.73534 (13)	−0.14402 (12)	0.0209 (3)	
N3	0.11031 (14)	0.56315 (13)	0.07218 (12)	0.0234 (4)	
C1	0.42582 (17)	0.87382 (17)	−0.16003 (15)	0.0251 (4)	
C2	0.41947 (19)	1.00119 (18)	−0.14135 (18)	0.0349 (5)	
H2A	0.5045	1.0180	−0.1440	0.052*	
H2B	0.3749	1.0100	−0.0668	0.052*	
H2C	0.3749	1.0573	−0.2012	0.052*	
C3	0.49426 (19)	0.8607 (2)	−0.27671 (17)	0.0383 (5)	
H3A	0.4992	0.7788	−0.2886	0.057*	
H3B	0.5790	0.8786	−0.2803	0.057*	
H3C	0.4485	0.9162	−0.3362	0.057*	
C4	0.4974 (2)	0.78700 (19)	−0.06747 (19)	0.0385 (5)	
H4A	0.5025	0.7051	−0.0795	0.058*	
H4B	0.4536	0.7952	0.0075	0.058*	
H4C	0.5821	0.8051	−0.0711	0.058*	
C5	0.29479 (17)	0.84524 (16)	−0.15391 (14)	0.0215 (4)	
C6	0.17783 (17)	0.91988 (16)	−0.15335 (14)	0.0231 (4)	
H6	0.1627	1.0038	−0.1566	0.028*	
C7	0.08916 (17)	0.84814 (16)	−0.14709 (14)	0.0213 (4)	
C8	−0.05270 (17)	0.88116 (16)	−0.14342 (15)	0.0240 (4)	
C9	−0.11551 (18)	0.82093 (17)	−0.03081 (16)	0.0282 (4)	
H9A	−0.0978	0.7342	−0.0259	0.042*	
H9B	−0.2062	0.8483	−0.0280	0.042*	
H9C	−0.0826	0.8419	0.0333	0.042*	
C10	−0.10238 (19)	0.84830 (18)	−0.24532 (16)	0.0323 (5)	
H10A	−0.0657	0.8911	−0.3166	0.048*	
H10B	−0.1937	0.8707	−0.2408	0.048*	
H10C	−0.0793	0.7622	−0.2434	0.048*	
C11	−0.08922 (18)	1.01676 (16)	−0.15143 (16)	0.0284 (4)	
H11A	−0.0619	1.0402	−0.0859	0.043*	
H11B	−0.1803	1.0394	−0.1516	0.043*	
H11C	−0.0487	1.0573	−0.2220	0.043*	
C12	0.11597 (17)	0.62036 (16)	−0.13713 (15)	0.0236 (4)	
H12A	0.1468	0.5897	−0.2084	0.028*	
H12B	0.0234	0.6312	−0.1297	0.028*	
C13	0.16583 (18)	0.52953 (16)	−0.03632 (15)	0.0237 (4)	
H13A	0.1469	0.4504	−0.0402	0.028*	
H13B	0.2579	0.5231	−0.0407	0.028*	
C14	0.17833 (17)	0.60915 (15)	0.12313 (15)	0.0224 (4)	
H14	0.2602	0.6181	0.0892	0.027*	
C15	0.13623 (17)	0.64936 (15)	0.23260 (14)	0.0213 (4)	
C16	0.00919 (17)	0.66223 (15)	0.27274 (15)	0.0224 (4)	
H16	−0.0489	0.6422	0.2303	0.027*	
C17	−0.03316 (17)	0.70369 (15)	0.37326 (14)	0.0219 (4)	
C18	0.05555 (17)	0.73581 (16)	0.43085 (15)	0.0225 (4)	
H18	0.0270	0.7671	0.4985	0.027*	
C19	0.18307 (17)	0.72475 (15)	0.39523 (14)	0.0217 (4)	
C20	0.22212 (16)	0.67702 (15)	0.29574 (14)	0.0209 (4)	
C21	−0.17007 (17)	0.71489 (16)	0.42364 (15)	0.0251 (4)	
C22	−0.22034 (19)	0.84395 (17)	0.44064 (17)	0.0315 (5)	
H22A	−0.1704	0.8656	0.4931	0.047*	
H22B	−0.3079	0.8503	0.4725	0.047*	
H22C	−0.2147	0.8982	0.3671	0.047*	
C23	−0.25348 (19)	0.68147 (19)	0.34618 (18)	0.0347 (5)	
H23A	−0.2492	0.7341	0.2717	0.052*	
H23B	−0.3401	0.6903	0.3807	0.052*	
H23C	−0.2245	0.5985	0.3366	0.052*	
C24	−0.1774 (2)	0.63108 (19)	0.53977 (17)	0.0358 (5)	
H24A	−0.1479	0.5485	0.5294	0.054*	
H24B	−0.2643	0.6393	0.5737	0.054*	
H24C	−0.1247	0.6520	0.5903	0.054*	
C25	0.27425 (17)	0.76568 (16)	0.46157 (15)	0.0237 (4)	
C26	0.20450 (19)	0.82364 (19)	0.56031 (16)	0.0319 (5)	
H26A	0.1424	0.8919	0.5307	0.048*	
H26B	0.2643	0.8509	0.5993	0.048*	
H26C	0.1622	0.7652	0.6142	0.048*	
C27	0.36982 (19)	0.65946 (17)	0.51248 (17)	0.0314 (5)	
H27A	0.3256	0.5969	0.5571	0.047*	
H27B	0.4197	0.6857	0.5620	0.047*	
H27C	0.4251	0.6280	0.4508	0.047*	
C28	0.34370 (19)	0.85899 (17)	0.38318 (16)	0.0305 (5)	
H28A	0.3907	0.8244	0.3193	0.046*	
H28B	0.4017	0.8834	0.4263	0.046*	
H28C	0.2830	0.9287	0.3539	0.046*	
C29	0.40841 (17)	0.53699 (16)	0.26496 (16)	0.0256 (4)	
H29A	0.3571	0.4911	0.2341	0.031*	
H29B	0.4186	0.5006	0.3457	0.031*	
C30	0.53345 (19)	0.53526 (18)	0.19799 (18)	0.0356 (5)	
H30A	0.5220	0.5677	0.1175	0.053*	
H30B	0.5784	0.4530	0.2055	0.053*	
H30C	0.5820	0.5840	0.2270	0.053*	

### Atomic displacement parameters (Å^2^)

**Table:**

	*U*^11^	*U*^22^	*U*^33^	*U*^12^	*U*^13^	*U*^23^
O1	0.0171 (7)	0.0243 (7)	0.0286 (7)	−0.0024 (6)	−0.0019 (5)	−0.0048 (5)
N1	0.0190 (8)	0.0283 (9)	0.0239 (8)	−0.0067 (7)	−0.0030 (6)	−0.0052 (6)
N2	0.0178 (8)	0.0230 (8)	0.0237 (8)	−0.0061 (7)	−0.0033 (6)	−0.0049 (6)
N3	0.0226 (9)	0.0247 (8)	0.0239 (8)	−0.0065 (7)	−0.0013 (6)	−0.0050 (6)
C1	0.0209 (11)	0.0286 (10)	0.0284 (10)	−0.0074 (9)	−0.0053 (8)	−0.0059 (8)
C2	0.0281 (12)	0.0329 (11)	0.0478 (13)	−0.0120 (10)	−0.0061 (9)	−0.0089 (9)
C3	0.0272 (12)	0.0542 (14)	0.0397 (12)	−0.0167 (11)	0.0002 (9)	−0.0158 (10)
C4	0.0327 (13)	0.0399 (12)	0.0457 (13)	−0.0115 (11)	−0.0180 (10)	−0.0003 (10)
C5	0.0230 (11)	0.0235 (9)	0.0196 (9)	−0.0067 (9)	−0.0042 (7)	−0.0036 (7)
C6	0.0243 (11)	0.0229 (9)	0.0235 (10)	−0.0040 (9)	−0.0041 (7)	−0.0064 (7)
C7	0.0225 (10)	0.0256 (9)	0.0166 (9)	−0.0042 (9)	−0.0030 (7)	−0.0048 (7)
C8	0.0211 (10)	0.0274 (10)	0.0254 (10)	−0.0043 (9)	−0.0047 (8)	−0.0074 (8)
C9	0.0223 (11)	0.0288 (10)	0.0341 (11)	−0.0068 (9)	0.0022 (8)	−0.0081 (8)
C10	0.0255 (11)	0.0399 (12)	0.0344 (11)	−0.0020 (10)	−0.0108 (9)	−0.0121 (9)
C11	0.0254 (11)	0.0284 (10)	0.0309 (10)	−0.0029 (9)	−0.0051 (8)	−0.0045 (8)
C12	0.0224 (10)	0.0267 (10)	0.0249 (9)	−0.0072 (9)	−0.0026 (7)	−0.0092 (8)
C13	0.0247 (11)	0.0240 (9)	0.0248 (10)	−0.0074 (9)	−0.0015 (8)	−0.0072 (7)
C14	0.0200 (10)	0.0236 (9)	0.0231 (9)	−0.0043 (8)	−0.0034 (7)	−0.0019 (7)
C15	0.0229 (10)	0.0201 (9)	0.0203 (9)	−0.0046 (8)	−0.0031 (7)	−0.0009 (7)
C16	0.0213 (10)	0.0234 (9)	0.0233 (9)	−0.0047 (8)	−0.0059 (7)	−0.0032 (7)
C17	0.0208 (10)	0.0204 (9)	0.0229 (9)	−0.0034 (8)	−0.0020 (7)	−0.0005 (7)
C18	0.0255 (11)	0.0231 (9)	0.0180 (9)	−0.0029 (8)	−0.0011 (7)	−0.0030 (7)
C19	0.0219 (10)	0.0213 (9)	0.0209 (9)	−0.0030 (8)	−0.0052 (7)	−0.0001 (7)
C20	0.0170 (10)	0.0210 (9)	0.0230 (9)	−0.0033 (8)	−0.0014 (7)	0.0000 (7)
C21	0.0203 (10)	0.0274 (10)	0.0279 (10)	−0.0056 (9)	−0.0012 (8)	−0.0048 (8)
C22	0.0263 (11)	0.0330 (11)	0.0345 (11)	−0.0031 (9)	−0.0006 (8)	−0.0080 (9)
C23	0.0217 (11)	0.0430 (12)	0.0424 (12)	−0.0084 (10)	−0.0014 (9)	−0.0127 (10)
C24	0.0320 (12)	0.0361 (11)	0.0368 (12)	−0.0103 (10)	0.0040 (9)	−0.0008 (9)
C25	0.0225 (11)	0.0268 (10)	0.0225 (9)	−0.0040 (9)	−0.0065 (7)	−0.0037 (7)
C26	0.0294 (12)	0.0404 (12)	0.0300 (11)	−0.0088 (10)	−0.0072 (8)	−0.0104 (9)
C27	0.0307 (12)	0.0326 (11)	0.0325 (11)	−0.0057 (10)	−0.0142 (9)	−0.0020 (8)
C28	0.0311 (12)	0.0323 (11)	0.0315 (11)	−0.0115 (10)	−0.0079 (9)	−0.0048 (8)
C29	0.0249 (11)	0.0248 (10)	0.0269 (10)	−0.0020 (9)	−0.0043 (8)	−0.0050 (8)
C30	0.0296 (12)	0.0330 (11)	0.0402 (12)	−0.0002 (10)	0.0028 (9)	−0.0052 (9)

### Geometric parameters (Å, º)

**Table:**

O1—C20	1.390 (2)	C14—C15	1.475 (2)
O1—C29	1.445 (2)	C14—H14	0.9500
N1—C5	1.333 (2)	C15—C20	1.396 (2)
N1—N2	1.3663 (19)	C15—C16	1.401 (2)
N2—C7	1.365 (2)	C16—C17	1.384 (2)
N2—C12	1.459 (2)	C16—H16	0.9500
N3—C14	1.268 (2)	C17—C18	1.402 (2)
N3—C13	1.463 (2)	C17—C21	1.533 (2)
C1—C5	1.519 (2)	C18—C19	1.394 (2)
C1—C3	1.527 (3)	C18—H18	0.9500
C1—C2	1.527 (3)	C19—C20	1.403 (2)
C1—C4	1.530 (3)	C19—C25	1.544 (2)
C2—H2A	0.9800	C21—C23	1.530 (3)
C2—H2B	0.9800	C21—C24	1.537 (3)
C2—H2C	0.9800	C21—C22	1.540 (3)
C3—H3A	0.9800	C22—H22A	0.9800
C3—H3B	0.9800	C22—H22B	0.9800
C3—H3C	0.9800	C22—H22C	0.9800
C4—H4A	0.9800	C23—H23A	0.9800
C4—H4B	0.9800	C23—H23B	0.9800
C4—H4C	0.9800	C23—H23C	0.9800
C5—C6	1.402 (3)	C24—H24A	0.9800
C6—C7	1.377 (2)	C24—H24B	0.9800
C6—H6	0.9500	C24—H24C	0.9800
C7—C8	1.522 (3)	C25—C26	1.531 (3)
C8—C11	1.536 (3)	C25—C28	1.536 (3)
C8—C9	1.537 (3)	C25—C27	1.536 (3)
C8—C10	1.541 (2)	C26—H26A	0.9800
C9—H9A	0.9800	C26—H26B	0.9800
C9—H9B	0.9800	C26—H26C	0.9800
C9—H9C	0.9800	C27—H27A	0.9800
C10—H10A	0.9800	C27—H27B	0.9800
C10—H10B	0.9800	C27—H27C	0.9800
C10—H10C	0.9800	C28—H28A	0.9800
C11—H11A	0.9800	C28—H28B	0.9800
C11—H11B	0.9800	C28—H28C	0.9800
C11—H11C	0.9800	C29—C30	1.495 (3)
C12—C13	1.522 (2)	C29—H29A	0.9900
C12—H12A	0.9900	C29—H29B	0.9900
C12—H12B	0.9900	C30—H30A	0.9800
C13—H13A	0.9900	C30—H30B	0.9800
C13—H13B	0.9900	C30—H30C	0.9800
			
C20—O1—C29	115.07 (13)	C20—C15—C16	119.59 (16)
C5—N1—N2	105.26 (14)	C20—C15—C14	120.33 (16)
C7—N2—N1	112.10 (14)	C16—C15—C14	120.07 (16)
C7—N2—C12	132.37 (15)	C17—C16—C15	121.13 (16)
N1—N2—C12	115.52 (14)	C17—C16—H16	119.4
C14—N3—C13	116.03 (15)	C15—C16—H16	119.4
C5—C1—C3	109.60 (15)	C16—C17—C18	117.23 (17)
C5—C1—C2	110.26 (16)	C16—C17—C21	123.37 (16)
C3—C1—C2	109.32 (16)	C18—C17—C21	119.40 (16)
C5—C1—C4	109.16 (15)	C19—C18—C17	124.18 (16)
C3—C1—C4	109.32 (17)	C19—C18—H18	117.9
C2—C1—C4	109.16 (16)	C17—C18—H18	117.9
C1—C2—H2A	109.5	C18—C19—C20	116.34 (16)
C1—C2—H2B	109.5	C18—C19—C25	121.02 (16)
H2A—C2—H2B	109.5	C20—C19—C25	122.64 (16)
C1—C2—H2C	109.5	O1—C20—C15	118.59 (16)
H2A—C2—H2C	109.5	O1—C20—C19	120.00 (15)
H2B—C2—H2C	109.5	C15—C20—C19	121.38 (17)
C1—C3—H3A	109.5	C23—C21—C17	111.74 (15)
C1—C3—H3B	109.5	C23—C21—C24	108.76 (16)
H3A—C3—H3B	109.5	C17—C21—C24	108.86 (15)
C1—C3—H3C	109.5	C23—C21—C22	108.38 (16)
H3A—C3—H3C	109.5	C17—C21—C22	110.09 (15)
H3B—C3—H3C	109.5	C24—C21—C22	108.96 (16)
C1—C4—H4A	109.5	C21—C22—H22A	109.5
C1—C4—H4B	109.5	C21—C22—H22B	109.5
H4A—C4—H4B	109.5	H22A—C22—H22B	109.5
C1—C4—H4C	109.5	C21—C22—H22C	109.5
H4A—C4—H4C	109.5	H22A—C22—H22C	109.5
H4B—C4—H4C	109.5	H22B—C22—H22C	109.5
N1—C5—C6	110.44 (15)	C21—C23—H23A	109.5
N1—C5—C1	118.91 (16)	C21—C23—H23B	109.5
C6—C5—C1	130.64 (16)	H23A—C23—H23B	109.5
C7—C6—C5	106.78 (16)	C21—C23—H23C	109.5
C7—C6—H6	126.6	H23A—C23—H23C	109.5
C5—C6—H6	126.6	H23B—C23—H23C	109.5
N2—C7—C6	105.42 (15)	C21—C24—H24A	109.5
N2—C7—C8	124.98 (15)	C21—C24—H24B	109.5
C6—C7—C8	129.60 (16)	H24A—C24—H24B	109.5
C7—C8—C11	108.78 (15)	C21—C24—H24C	109.5
C7—C8—C9	111.03 (15)	H24A—C24—H24C	109.5
C11—C8—C9	107.95 (15)	H24B—C24—H24C	109.5
C7—C8—C10	111.28 (15)	C26—C25—C28	107.19 (16)
C11—C8—C10	107.43 (15)	C26—C25—C27	107.37 (15)
C9—C8—C10	110.24 (15)	C28—C25—C27	109.42 (16)
C8—C9—H9A	109.5	C26—C25—C19	111.32 (15)
C8—C9—H9B	109.5	C28—C25—C19	110.49 (14)
H9A—C9—H9B	109.5	C27—C25—C19	110.93 (15)
C8—C9—H9C	109.5	C25—C26—H26A	109.5
H9A—C9—H9C	109.5	C25—C26—H26B	109.5
H9B—C9—H9C	109.5	H26A—C26—H26B	109.5
C8—C10—H10A	109.5	C25—C26—H26C	109.5
C8—C10—H10B	109.5	H26A—C26—H26C	109.5
H10A—C10—H10B	109.5	H26B—C26—H26C	109.5
C8—C10—H10C	109.5	C25—C27—H27A	109.5
H10A—C10—H10C	109.5	C25—C27—H27B	109.5
H10B—C10—H10C	109.5	H27A—C27—H27B	109.5
C8—C11—H11A	109.5	C25—C27—H27C	109.5
C8—C11—H11B	109.5	H27A—C27—H27C	109.5
H11A—C11—H11B	109.5	H27B—C27—H27C	109.5
C8—C11—H11C	109.5	C25—C28—H28A	109.5
H11A—C11—H11C	109.5	C25—C28—H28B	109.5
H11B—C11—H11C	109.5	H28A—C28—H28B	109.5
N2—C12—C13	111.74 (14)	C25—C28—H28C	109.5
N2—C12—H12A	109.3	H28A—C28—H28C	109.5
C13—C12—H12A	109.3	H28B—C28—H28C	109.5
N2—C12—H12B	109.3	O1—C29—C30	107.58 (15)
C13—C12—H12B	109.3	O1—C29—H29A	110.2
H12A—C12—H12B	107.9	C30—C29—H29A	110.2
N3—C13—C12	111.54 (15)	O1—C29—H29B	110.2
N3—C13—H13A	109.3	C30—C29—H29B	110.2
C12—C13—H13A	109.3	H29A—C29—H29B	108.5
N3—C13—H13B	109.3	C29—C30—H30A	109.5
C12—C13—H13B	109.3	C29—C30—H30B	109.5
H13A—C13—H13B	108.0	H30A—C30—H30B	109.5
N3—C14—C15	123.02 (17)	C29—C30—H30C	109.5
N3—C14—H14	118.5	H30A—C30—H30C	109.5
C15—C14—H14	118.5	H30B—C30—H30C	109.5
			
C5—N1—N2—C7	0.53 (18)	C20—C15—C16—C17	1.0 (3)
C5—N1—N2—C12	−179.65 (14)	C14—C15—C16—C17	−177.78 (16)
N2—N1—C5—C6	−0.46 (18)	C15—C16—C17—C18	2.0 (2)
N2—N1—C5—C1	179.95 (14)	C15—C16—C17—C21	−177.51 (16)
C3—C1—C5—N1	70.9 (2)	C16—C17—C18—C19	−2.2 (3)
C2—C1—C5—N1	−168.71 (16)	C21—C17—C18—C19	177.33 (16)
C4—C1—C5—N1	−48.8 (2)	C17—C18—C19—C20	−0.7 (3)
C3—C1—C5—C6	−108.6 (2)	C17—C18—C19—C25	178.16 (16)
C2—C1—C5—C6	11.8 (3)	C29—O1—C20—C15	−71.21 (19)
C4—C1—C5—C6	131.7 (2)	C29—O1—C20—C19	110.92 (17)
N1—C5—C6—C7	0.2 (2)	C16—C15—C20—O1	178.06 (15)
C1—C5—C6—C7	179.77 (17)	C14—C15—C20—O1	−3.2 (2)
N1—N2—C7—C6	−0.38 (19)	C16—C15—C20—C19	−4.1 (3)
C12—N2—C7—C6	179.83 (16)	C14—C15—C20—C19	174.68 (16)
N1—N2—C7—C8	179.90 (14)	C18—C19—C20—O1	−178.33 (15)
C12—N2—C7—C8	0.1 (3)	C25—C19—C20—O1	2.9 (2)
C5—C6—C7—N2	0.09 (19)	C18—C19—C20—C15	3.9 (2)
C5—C6—C7—C8	179.79 (16)	C25—C19—C20—C15	−174.95 (16)
N2—C7—C8—C11	−178.72 (16)	C16—C17—C21—C23	−3.2 (2)
C6—C7—C8—C11	1.6 (2)	C18—C17—C21—C23	177.30 (16)
N2—C7—C8—C9	62.6 (2)	C16—C17—C21—C24	116.99 (19)
C6—C7—C8—C9	−117.0 (2)	C18—C17—C21—C24	−62.6 (2)
N2—C7—C8—C10	−60.6 (2)	C16—C17—C21—C22	−123.64 (18)
C6—C7—C8—C10	119.8 (2)	C18—C17—C21—C22	56.8 (2)
C7—N2—C12—C13	−124.46 (19)	C18—C19—C25—C26	−3.6 (2)
N1—N2—C12—C13	55.76 (19)	C20—C19—C25—C26	175.12 (16)
C14—N3—C13—C12	−102.04 (18)	C18—C19—C25—C28	−122.60 (18)
N2—C12—C13—N3	66.26 (19)	C20—C19—C25—C28	56.1 (2)
C13—N3—C14—C15	179.25 (16)	C18—C19—C25—C27	115.87 (19)
N3—C14—C15—C20	167.22 (17)	C20—C19—C25—C27	−65.4 (2)
N3—C14—C15—C16	−14.0 (3)	C20—O1—C29—C30	168.72 (15)
